# The Association of Lipoprotein Lipase Genes, HindIII and S447X Polymorphisms With Coronary Artery Disease in Shiraz City

**DOI:** 10.15171/jcvtr.2015.14

**Published:** 2015

**Authors:** Zeinab Ahmadi, Sara Senemar, Samaneh Toosi, Salma Radmanesh

**Affiliations:** ^1^ Human Genetics Research Group, Iranian Academic Center for Education Culture & Research (ACECR), Fars Branch, Shiraz, Iran; ^2^ Department of Cardiology, Shiraz University of Medical Sciences, Shiraz, Iran

**Keywords:** Coronary Artery Disease, Lipoprotein Lipase Mutations, Smoking, Hypertension, Hyperlipidemia, Triglycerides, S447X, HindIII

## Abstract

***Introduction:*** Several polymorphisms at the lipoprotein lipase (LPL) locus are associated with variations in LPL activity serum lipid concentrations and the risk of coronary artery disease (CAD). The aim of this study was to investigate the role of the LPL S447X and HindIII polymorphism in a sample of subjects with CAD and compare them with healthy subjects.

***Methods:*** The study enrolled 115 patients and 89 healthy subjects who were recruited from Namazi hospital in 2010-2012. The presence of two common polymorphisms of the LPL gene (HindIII and S447X) was determined by polymerase chain reaction restriction fragment length polymorphism (PCR-RFLP) analysis using genomic DNA. SPSS 16.0 was used for statistical analysis.

***Results:*** S447X was significantly different between the patients with CAD and the healthy subjects (*P* < 0.001). But HindIII was not significantly different between the patients with CAD and the healthy subjects (*P* = 0.741). Risk factors such as smoking, hypertension, hyperlipidemia, triglyceride (TG) and high-density lipoprotein (HDL) levels had a significant association with CAD.

***Conclusion:*** In our study, the presence of G allele S447X polymorphism increases the TG level and decrease HDL level, so it increases the susceptibility CAD. Moreover, HindIII polymorphism did not have any significant association with CAD.

## Introduction

Lipoprotein lipase (LPL) found in 1943. This enzyme plays a central role in lipid metabolism by hydrolyzing triglyceride-rich particles in the muscles. Free fatty acids and glycerol have been produced by adipose tissue and macrophages for energy utilization and storage. The LPL gene is located on chromosome 8p22.^1^ 100 mutations have been described in this gene. Several polymorphisms at the LPL locus are associated with variations in LPL activity serum lipid concentrations and the risk of coronary artery disease (CAD).^2^ In most previous studies Ser447Stop polymorphism has enhanced LPL activity and has been resulted to be related to decreased TG and increased HDL-C.^3-5^ Furthermore HindIII polymorphism has been resulted to be associated with elevated TG, lower HDL-C, and an increased risk of CAD in most studies.^3,6,7^ The aim of this study was to investigate the role of the LPL S447X and HindIII polymorphism in a sample of subjects with CAD and compare them with healthy controls.

## Materials and Methods

### Study Population

The study population comprised 115 patients (72 males and 43 females, aged 31-55, mean age 46.6±4.96 year) and 89 control subjects (45 males and 44 females, aged 22-67, mean age 48.8±9.64) who underwent coronary angiography because of either symptoms of suspected CAD or unrelated conditions such as cardiomyopathy or valvular heart disease. Positive angiography was defined as the presence of greater than 50% coronary diameter reduction as described by an experienced cardiologist, while control subjects were those who had <30% stenosis in all major vessels. All participants were apprised on the aims of the study and informed written consent was obtained from them. Study was approved by the ethics committee of Shiraz Medical University. Each participant was interviewed by two trained interviewers about the cardiovascular risk factors such as hypertension (defined as systolic/diastolic blood pressures higher than 140/90 mm Hg or using antihypertensive medications), hyperlipidemia, TG and HDL levels and cigarette smoking habit (cigarette and/or hookah). The subjects who smoked at least one cigarette or hookah per day or were ex-smokers were considered as smokers while non-smokers were defined as those who had never smoked. All subjects in this study were recruited from Namazi hospital between July 2010 and March 2012. Sample size was estimated upon primary study by MedCalc software with α=0.05 and 1-β=%80.

### DNA Isolation and Polymorphism Analysis

Genomic DNA for polymerase chain reaction (PCR) was isolated from the whole blood by using salting out methods. Polymerase chain reaction restriction fragment length polymorphism (PCR-RFLP) determined the presence of two common polymorphisms of the LPL gene. The primer sets and procedures were selected from the published information as follows^4,8^:

HindIII forward primer: 5’-TGAAGCTCAAATGGAAGAGT-3’, and reverse primer: 5’-TACAAGCAAATGACTAAA-3’;

S447X forward primer: 5’-TACACTAGCAATGTCT AGCTGAAGGCAGA -3’, and reverse primer: 5’-TCAG CTTTAGCCCAGAATGCTCACC-3’.

The MnLI restriction endonuclease (Fermentase) was used to detect Ser447Stop polymorphism. Homozygote of the mutation was designated (GG), that of the wild type was designated (CC), and heterozygote was also designated (GC). Also HindIII polymorphism was detected by digestion of PCR products with HindIII restriction endonuclease (Fermentase). Homozygote of the mutation was designated H-H-, that of the wild type was designated H^+^H^+^, and heterozygote was designated H^+^H-.^3,9^

### Statistical Analysis

Gene-Counting was used to calculate the frequencies of genotypes and alleles. SPSS 16.0 was used for statistical analysis. Comparisons of groups were performed using chi-square test. The binary logistic test was also used to further estimate the association between the polymorphism and the developing risk of CAD. *P*-value less than 0.05 was considered significant.

## Results

The distribution of the HindIII genotype in the whole population was compatible with Hardy-Weinberg proportion (*P*=0.47), with allele frequencies of 77% and 23% for the H^+^ and H-, respectively (Table 1). Also the distribution of the S447X genotype in the whole population was not compatible with Hardy-Weinberg proportion (*P*<0.001), with allele frequencies of 73% and 27% for the C and G, respectively (Table 1). Likewise as Table 2 shows the genotype distribution and allele frequencies for S447X and HindIII gene between CAD patients and control subjects. As shown in Table 2, S447X was significantly different between the patients with CAD and the control subjects (*P*<0.001). But HindIII was not significantly different between the patients with CAD and the control subjects (*P*=0.741).

**Table 1:** Hardy-Weinberg Equilibrium Test of HindIII and S447x of LPL Gene

**Genotype**	**Observed count**	**Expected count**	**Genotype frequency (%)**	**Allele**	**Observed count**	**Allele frequencies**	**X** ^ 2 ^	***P***
HindIII								
H^+^H^+^	114	116.80	57.87	H^+^	302	0.77	0.524	0.47
H^+^H-	74	69.78	37.56	H-	92	0.23		
H-H-	9	10.42	4.57					
Total	197	197	100.00		394			
S447X								
CC	133	108.71	65.20	C	296	0.73	82.96	<0.001
CG	30	80.42	14.70	G	112	0.23		
GG	41	14.87	20.10					
Total	204	204	100.00		408			

**Table 2:** Allelic and Genotypic Frequencies as Well as Association of S447X Polymorphism and CAD

**S447X polymorphism**	**Control (n=89)**	**Case (n=115)**	*** P ***	**OR**	**95% CI**
CC	75 (84.3%)	58 (50.4%)	<0.001		
CG	7 (7.9%)	23 (20.0%)			
GG	7 (7.9%)	34 (29.6%)			
HindIII polymorphism	Control (n=89)	Case (n=108)			
H^+^H^+^	53 (59.6%)	61 (56.5%)	0.741		
H^+^H-	33 (37.1%)	41 (38.0%)			
H-H-	3 (3.4%)	6 (5.6%)			
Allele frequency					
C	157 (88.2%)	139 (60.4%)	<0.001		
G	21 (11.8%)	91 (39.6%)			
H^+^	139 (78.1%)	163 (75.5%)	0.540		
H-	39 (21.9%)	53 (24.5%)			
S447X polymorphism	Control (n=89)	Case (n=115)			
CC	75 (84.3%)	58 (50.4%)			
CG + GG	14 (15.7%)	57 (49.6%)	<0.001	5.265	2.673-10.368

Demographic characteristics of the participants are summarized in Table 3. The results presented in Table 3 indicate that these risk factors had a significant association with CAD (smoking: OR=3.256, *P*<0.001; hypertension: OR=1.952, *P*=0.025; hyperlipidemia: OR=2.347, *P*=0.004; TG>200 mg/dl: OR=2.345, *P*=0.004; HDL<40 mg/dl: OR=1.917, *P*=0.024). As shown in Table 2, S447X polymorphism had a significant association with CAD. Moreover, CG+GG genotype was an additive risk factor for CAD (OR=5.265, *P*<0.001). The interaction between S447X polymorphism and TG and HDL-C with CAD is shown in Table 4. The interaction between polymorphism S447X and demographic risk factors (hyperlipidemia, hypertension, and smoking) separately with CAD is shown in Table 5. The results indicate that there was a significant association with CAD.

**Table 3:** Risk Factors for CAD in Patients and Controls

	**Control (%)**	**Case (%)**	***P***	**OR**	**95% CI**
Smoking					
No	66 (74.2)	52 (46.8)			
Yes	23 (25.8)	59 (53.2)	<0.001	3.256	1.781-5.953
Hypertension					
No	62 (69.7)	60 (54.1)			
Yes	27 (30.3)	51 (45.9)	0.025	1.952	1.086-3.508
Hyperlipidemia					
No	60 (67.4)	52 (46.8)			
Yes	29 (32.6)	59 (53.2)	0.004	2.347	1.315-4.189
TG					
<200 mg/dl	59 (66.3)	52 (45.6)			
>200 mg/dl	30 (33.7)	62 (54.4)	0.004	2.345	1.321-4.161
HDL					
>40 mg/dl	56 (62.9)	54 (47.0)			
<40 mg/dl	33 (37.1)	61 (53.0)	0.024	1.917	1.090-3.372

**Table 4:** Association of S447X Polymorphism and TG and HDL Levels With CAD

**Polymorphism S447X**	**TG (mg/dl)**	**HDL (mg/dl)**	**Control (%)**	**Case (%)**	**P-value**	**OR**	**95% CI**
CC	<200	>40	40 (44.9)	17 (14.9)	<0.001	-	-
		<40	11 (12.4)	8 (7.0)	0.326	1.711	0.585-5.004
	>200	>40	9 (10.1)	12 (10.5)	0.030	3.137	1.116-8.822
		<40	15 (16.9)	20 (17.6)	0.011	3.137	1.304-7.545
CG + GG	<200	>40	6(6.7)	20 (17.6)	<0.001	7.843	2.678-22.966
		<40	2 (2.3)	7 (6.1)	0.013	8.235	1.549-43.782
	>200	>40	1 (1.1)	4 (3.5)	0.052	9.412	0.979-90.518
		<40	5 (5.6)	26 (22.8)	<0.001	12.235	4.021-37.226

**Table 5:** Association of S447X Polymorphism and Demographic Risk Factors With CAD

**Polymorphism**	**Risk factors**	**Control (%)**	**Case (%)**	***P***	**OR**	**95% CI**
S447X	Hyperlipidemia					
CC	No	52 (58.4)	26 (23.4)	<0.001	-	-
	Yes	23 (25.8)	30 (27.0)	0.009	2.609	1.271-5.353
CG + GG	No	8 (9.0)	26 (23.4)	<0.001	6.500	2.586-16.338
	Yes	6 (6.8)	29 (26.2)	<0.001	9.667	3.566-26.202
S447X	Hypertension					
CC	No	53 (59.6)	26 (24.1)	<0.001	-	-
	Yes	22 (24.7)	27 (25.0)	0.014	2.502	1.202-5.206
CG + GG	No	9 (10.1)	31 (28.7)	<0.001	7.021	2.918-16.896
	Yes	5 (5.6)	24 (22.2)	<0.001	9.785	3.350-28.575
S447X	Smoking					
CC	No	55 (61.8)	31 (27.9)	<0.001	-	-
	Yes	20 (22.5)	25 (22.5)	0.034	2.218	1.064-4.623
CG + GG	No	11 (12.3)	21 (18.9)	0.005	3.387	1.445-7.941
	Yes	3 (3.4)	34 (30.6)	<0.001	20.108	5.704-70.877

## Discussion

CAD is a complex disease with genetic and environmental components. The results of most genetic studies of CAD are different, perhaps because the genetic risk of CAD is not based on a single gene but is based on interactions among multiple genes and environmental risk factors. LPL is a potential target for treatment of CAD because LPL gene variants are involved in multiple genes and environmental risk factors associated with CAD.^10,11^ One of the most desirable candidate genes is LPL gene that may be explain some of the lipid and lipoprotein abnormalities faced in numerous cases of CAD.^12^ In this study, we presented the results on polymorphisms of the LPL gene, HindIII and S447X and their association with CAD and environmental risk factors including smoking, hypertension, hyperlipidemia, TG and HDL-C levels. To the best of our knowledge, it is the first study on the population of the south of Iran.

The frequency of H-allele HindIII polymorphism is 22%. Sayad et al.^13^ reported that the frequency of H- allele HindIII polymorphism was 30%. In a recent study, there was not any significant association between CAD and the control groups (*P*=0.540). Moreover, the frequency of G allele S447X polymorphism was 12% and there was an association between CAD and the control group (*P*<0.001). Therefore, we can conclude that S447X polymorphism is associated with CAD (*P*<0.001, OR=5.265, 95% CI= 2.673-10.368). The presence of G allele is due to increased susceptibility of CAD. In this study, we investigated the associations of environmental risk factors with CAD. We could observe that as previous studies, in this study, there were associations between demographic risk factors (smoking, hypertension, hyperlipidemia, TG> 200 mg/dl and HDL<40 mg/dl) and CAD.^2,9,12,14,15^ These risk factors were due to the increase in the susceptibility to CAD. Smoking had the most influence on this disease ( smoking: *P*<0.001, OR=3.256, 95% CI=1.781-5.953; hypertension: *P*=0.025, OR=1.952, 95% CI=1.086-3.508; hyperlipidemia: *P*=0.004, OR=2.347, 95% CI=1.315-4.189; TG>200 mg/dl: *P*=0.004, OR=2.345, 95% CI=1.321-4.161; HDL<40 mg/dl: *P*=0.024, OR=1.917, 95% CI=1.090-3.372).

Another analysis done in this study is the impact of each risk factor with S447X polymorphism. In all analyses, we could observe that the presence of G allele and environmental risk factor is due to the increased susceptibility to CAD.

About TG and HDL-C level, LPL gene has changed the TG and HDL measurement. We could study the influence of both risk factors. The result is G allele, increasing TG level and decreasing HDL level in the case group. Therefore, we conclude that S447X polymorphism influences the plasma lipid levels. In several studies, S447X polymorphism has played a protective role in CAD although in a recent study the result is different.^1,12,14,16^ Perhaps, S447X polymorphism has affected another function in our population that increases the risk for disease and did not have any protection effect.

In conclusion, in our study, the presence of G allele S447X polymorphism increases the TG level and decrease HDL level, so it increases the susceptibility CAD. Moreover, HindIII polymorphism doesn’t have any significant association with CAD.

The limitation of this study is the number of our groups. It is recommended that in future studies, the number of samples should be more than our study.

## Acknowledgments

The authors acknowledge financial support from human genetics research group of ACECR, Fars branch and the hospitals staff for helping us. The authors would like to thank Dr. Nasrin Shokrpour at Center for Development of Clinical Research of Nemazee Hospital for editorial assistance.

## Ethical Issues

All patients gave written informed consents and the study was approved by our local ethic committee.

## Conflict of Interests

There is no conflict of interest on this article.
